# Real world analysis on the efficacy and safety of anti-tumor necrosis factor therapy in patients with stricturing Crohn’s disease

**DOI:** 10.1038/s41598-021-90660-2

**Published:** 2021-06-03

**Authors:** Sudheer K. Vuyyuru, Bhaskar Kante, Peeyush Kumar, Pabitra Sahu, Saurabh Kedia, Mukesh Kumar Ranjan, Raju Sharma, Rajesh Panwar, Govind Makharia, Vineet Ahuja

**Affiliations:** 1 Department of Gastroenterology, All India Institute of Medical Sciences, Ansari Nagar, New Delh, 110029 India; 2 Department of Radiodiagnosis, All India Institute of Medical Sciences, New Delhi, India; 3 Department of Gastrointestinal Surgery and Liver Transplantation, All India Institute of Medical Sciences, New Delhi, India

**Keywords:** Inflammation, Anaemia, Crohn's disease

## Abstract

Crohn’s disease (CD) is often complicated by strictures and associated with increased risk for surgery. Inflammatory strictures respond to medical therapy, and anti-tumor necrosis factor (TNF) therapy is often used after the failure of steroids. However, data on efficacy of anti-TNF therapy in stricturing CD is limited. We retrospectively analysed the records of patients with stricturing CD who were treated with anti-TNF therapy and were prospectively followed from January 2005 to July 2020. Treatment success was defined as continuation of anti-TNF without the requirement for steroids or parenteral nutrition, switch to other anti-TNF, endoscopic dilation, surgery and severe adverse events leading to the withdrawal of anti-TNF. Fifty-nine patients were included [50-infliximab, 9-adalimumab; mean age-30.1 ± 15 years; males-69.5%; median disease duration-124 (range 30–396) months; median follow-up duration-42 (range 8–180) months]. Ileum was the most common site of stricture (69.5%), 20.3% of patients had colonic strictures, and 64.4% had multiple strictures. 55.9% of patients were steroid dependent and 37.3% were steroid refractory. The median duration of anti-TNF therapy was 14 (range 2–96) months, and 54.2% (n = 32) patients received concomitant immunomodulators. 88% improved with induction (11.8% primary non-response), secondary loss of response was seen in 52.2%, and the cumulative probability of treatment success at 1, 2 and 5 years was 69%, 51%, and 28% respectively. Anaemia at presentation predicted poor response. Only 30% of patients retained biologics on long-term (lack of response, cost, adverse events). 16.9% had adverse events, the commonest being reactivation of tuberculosis (5.1%). Anti-TNF therapy is associated with good short-term treatment success with modest long-term response in stricturing CD.

## Introduction

Crohn’s disease (CD) is an immune mediated inflammatory intestinal disease often complicated by strictures, fistulae and abscess. Though strictures can form early in the disease course as shown in the Epi-IBD inception cohort^1^, most patients develop these complications during the natural course of CD^2^, progressing with disease duration, as evident in population based cohort from Olmsted County, in which 19% patients had stricturing or penetrating complication at diagnosis or within 90 days of diagnosis, and by 20 years half the cohort experienced complications like strictures and fistulae^3^.

Three key components in defining stricture are bowel wall thickening, pre-stenotic dilatation and luminal narrowing, and the presence of any of these two components is suggestive of a stricture. Strictures can be formed de novo or at the anastomotic site after bowel resection. They may develop anywhere in the small bowel and large bowel but most common site is the ileum. Though there is no clear-cut demarcation, early in the disease course the strictures can be predominantly inflammatory early in the disease course, or predominantly fibrotic after anti-inflammatory therapies. The inflammatory component of strictures can be tackled with immunosuppressants whereas fibrotic strictures often require surgical intervention in the form of resection or stricturoplasty or endoscopic intervention like balloon dilatation or stricturotomy. These procedures relieve mechanical obstruction but do not affect the underlying fibrogenic process, which also has remained largely refractory to existing medical therapies.

Unlike luminal CD, patients with symptomatic stricturing CD have largely been excluded from the registration trials of most of the available therapies for CD, and there are no well-designed trials which have evaluated the efficacy of immunosuppressants like steroids, immunomodulators, and biologics in this group of patients. Hence, the evidence is mostly extrapolated from the trials on inflammatory CD or from the real-world retrospective or prospective cohort studies which have separately evaluated stricturing CD. Among the available maintenance therapies, though the immunomodulators like azathioprine and methotrexate have been found useful in limiting the progression of CD, they are considered ineffective as primary agents. Among the biologics, anti-TNF agents have the maximum evidence, however, this is also limited to a few retrospective studies and three prospective studies including the CREOLE study by Bouhnik et al., which evaluated the efficacy of adalimumab^4–10^ in 97 patients. The initial concern (progression of stricture, perforation) with the use of anti-TNF therapy in stricturing CD has gradually waned off with upcoming literature on their efficacy and safety, and the present study adds to this growing evidence. Role of anti-TNF therapy in prevention of surgery in stricturing CD is controversial. One predictive model (BACARDI) developed by Belgium group showed high risk of surgery with anti-TNF exposure^11^. Even though various retrospective studies of vedolizumab in CD included a significant fraction of patients with strictures and fistulae, data on stratified outcomes based on behaviour (inflammatory/stricturing/penetrating) are not available, and there are no data available for ustekinumab, and JAK inhibitors as well. Moreover, these newer agents are still not available in all the countries, especially the developing ones, where the disease burden of IBD is increasing. Hence, anti-TNF agents remain the medical therapy of choice for this subgroup of difficult to treat patients, and more evidence is required on their efficacy and safety in this situation. Hence, this retrospective cohort study was conducted to evaluate the efficacy and safety of anti-TNF therapy in patients with stricturing Crohn’s disease.

## Materials and methods

### Study population

This study included patients with stricturing CD who were treated with anti-TNF agents and were under follow-up at the inflammatory bowel disease (IBD) clinic, Department of Gastroenterology, All India Institute of Medical Sciences (AIIMS), New Delhi, India from January 2005 to July 2020.

### Study design and data collection

In this retrospective study, we collected data from prospectively maintained database of patients with inflammatory bowel disease. All patients with stricturing CD (including small bowel and large bowel strictures) treated with anti-TNF agents were included in this study. Data on demographic features, disease characteristics (including location, extent, severity, behavior), site of stricture, radiological details, treatment received, response to therapy, and long-term complications were recorded. Any missing data was confirmed by interviewing the patient in person.

### Ethical considerations

The study protocol was written in accordance with the ethical guidelines of the 1975 Declaration of Helsinki as reflected in a priori approval by the Institutional Ethics Committee (IEC) of All India Institute of Medical Sciences on 27.03.2019 (IRB No: IECPG-219/27.03.2019). As this is a retrospective study consent from participants has been waived off by the ethics committee.

### Definitions

#### Crohn’s disease

Diagnosis of CD was made as per European Crohn’s and Colitis Organization (ECCO) guidelines, based upon characteristic clinical, radiologic, endoscopic, and histologic features. Disease activity was measured by the CD activity index (CDAI)^12^. Disease location and behavior were classified based on Montreal classification^13^.

#### Definitions of stricture

A lesion with combination of a reduction of luminal narrowing > 50%, an increase in bowel wall thickness > 25% relative to non-affected bowel and pre-stricture dilation > 3 cm. For strictures identified during endoscopic procedure defined by luminal narrowing with inability to pass the endoscope across the narrowing^14^.

#### Steroid response

Decrease in Crohn’s disease activity index by ≥ 100 points without intestinal obstruction ^15^.

### Response to anti-TNF

Decrease in Crohn’s disease activity index by ≥ 100 points without intestinal obstruction.

### Treatment success

Continuation of anti-TNF with all the following criteria^5^

- no use of corticosteroids or parenteral nutrition, or switch to other anti-TNFs
- no endoscopic dilation
- no bowel surgery for resection of stricture
- no severe adverse events leading to the withdrawal of anti-TNF

### Primary non-response

Lack of initial clinical response to the index anti-TNF agent (decrease in CDAI by < 100 points) assessed at week 14 after initiation^16^.

### Secondary loss of response

Loss of response in patients with initial response to the anti-TNF agent^16^.

### Statistical analysis

Categorical variables were expressed as percentages and continuous variables were expressed mean ± SD or median (range) as appropriate. Chi-square test was used to compare categorical variables and Student t-test or Mann–Whitney U-test was used to compare continuous variables as appropriate. P < 0.05 was considered statistically significant. Survival analysis was done to evaluate the cumulative response with therapy. Predictors of response to anti-TNFs were estimated with Cox-proportional hazard model. After univariate analysis, all the variables with P values less than 0.15 were considered in the subsequent multivariate analysis. SPSS software version 21.0 (IBM Corp., Armonk, NY, USA) was used for statistical analysis.

### Ethical approval

Obtained (IRB No: IECPG-219/27.03.2019).

## Results

From January 2005 to July 2020, 224 patients of Crohn’s disease with strictures (B2) were identified. Among these patients, 59 patients received anti-TNF therapy (Infliximab and Adalimumab biosimilar) and were included in the final analysis. All patients with strictures and associated internal fistula were excluded from the analysis.

### Baseline characteristics

The mean age at presentation was 30.1 ± 15.6 years (A1: 27.1; A2: 40.7%; A3: 32.2%) and majority were male [69.5% (n = 41)]. The median duration of Crohn’s disease was 124 (range: 30–396) months and patients were followed up for a median duration of 42 (range: 8–180) months. 52.5% patients had abdominal pain, 42.4% had diarrhoea, 15.3% (n = 9) had associated perianal fistula, and 10.2% (n = 6) had history of subacute intestinal obstruction. Ileum was the most common site of stricture [69.5%, n = 41], colonic strictures were seen in 20.3% (n = 12) patients, and only 10.2% (n = 6) patients had jejunal strictures. 64.4% (n = 38) patients had more than one stricture. In only 45.8% (n = 27) patients, the stricture was accessible to endoscopy. Anaemia was seen in 59.3% (n = 35) patients and the mean haemoglobin of entire cohort was 9.3gm/dl (SD ± 2.0). 39% (n = 23) patients were considered for therapeutic ATT trial to confirm the diagnosis of Crohn’s disease before initiation of immunosuppressants. Extraintestinal manifestations were seen in 18.6% (n = 11). (Table 1).

**Table 1:** Baseline characteristics of patients with stricturing Crohn’s disease.

Parameter		N (%) (n = 59)
Age at presentation (Mean ± SD)		30.1 ± 15.6
A1		16 (27.1%)
A2		24 (40.7%)
A3		19 (32.2%)
Sex (%)		
Male		41 (69.5%)
Female		18 (30.5%)
Median disease duration of disease (range) (months)		124 (30–396)
Overall median duration of follow up (range) (months)		42 (8–180)
Median duration between disease onset and initiation of biologics (months)		72 (50–120)
Median duration of follow up after initiation of biologics (months)		26 (16–50)
Clinical symptoms		
Diarrhoea		25 (42.4%)
Abdominal pain		31 (52.5%)
Blood in stools		16 (27.1%)
Subacute intestinal obstruction		6 (10.2%)
Perianal fistula		9 (15.3%)
Anaemia at baseline		35 (59.3%)
Extra intestinal manifestations		11 (18.6%)
Disease location (L), n (%)	Terminal Ileal ± ileocaecal (L1)	13 (22.0%)
	Colonic (L2)	14 (23.7%)
	Ileocolonic (L3)	12 (20.3%)
	Upper gastrointestinal (L4)	13 (22.0%)
	L1 + 4 (with upper GI modifier)	6 (10.2%)
	L3 + 4 (with upper GI modifier)	1 (1.7%)
Location of stricture (%)	Ileal	41 (69.5%)
	Jejunal +/− Ileal	6 (10.2%)
	Colonic	12 (20.3%)
Number of strictures		
Single		21 (35.6%)
Multiple		38 (64.4%)
Stricture noted on endoscopy		27 (45.8%)
Baseline Haemoglobin (mean ± SD) (gm/dl)		9.3 ± 2.0
Baseline Albumin (mean ± SD) (gm/dl)		3.3 ± 0.85
Steroid dependent disease		33 (55.9%)
Steroid refractory disease		22 (37.3%)
≥ 3 courses of steroids		20 (33.9%)
Azathioprine received (overall)		52 (88.0%)
Concomitant immunomodulators		
Azathioprine		28 (47.8%)
Methotrexate		4 (6.8%)
Therapeutic ATT trial		23 (39.0%)

### Treatment outcomes

### Steroids and immunomodulators

A Majority (93.2%; n = 55) of the patients experienced at least one steroid course and 56% (n = 33) had a clinical response. 33.9% (n = 20) patients received three or more courses of steroids. 55.9% (n = 33) were steroid dependent and 37.3%% (n = 22) were refractory to steroid therapy. 88% (n = 52) patients experienced therapy with azathioprine, of which 55.9% (n = 33) were prescribed azathioprine as a form of primary therapy (before starting anti-TNF agents). However, response to azathioprine could be assessed in only 40.6% (n = 24) patients as rest received it for a duration of less than 6 months. Among these patients, 29% (7/24) maintained clinical remission induced by steroids for a median duration of 23 months. 47.5% (n = 28) patients received azathioprine along with anti-TNF therapy which was either started along with anti-TNF therapy (n = 22) or continued along with biologics if they had already been on azathioprine (n = 6). Nine patients received subcutaneous methotrexate (in five patients as monotherapy and in four patients as concomitant therapy).

### Anti-TNF therapy

**Figure 1:**
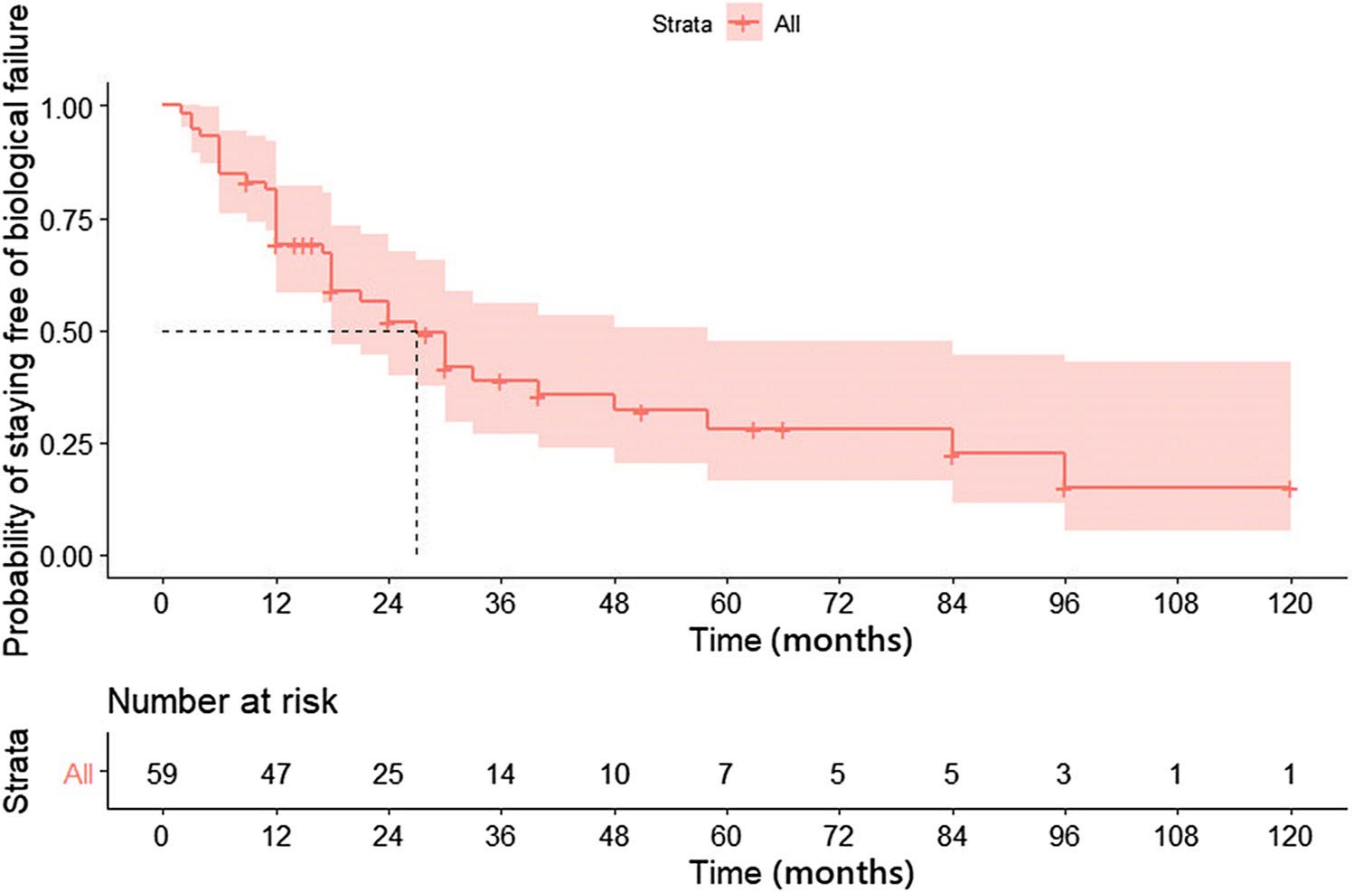
Kaplan Meier graph showing probability of anti TNF success in patients with stricturing Crohn’s disease.

**Figure 2:**
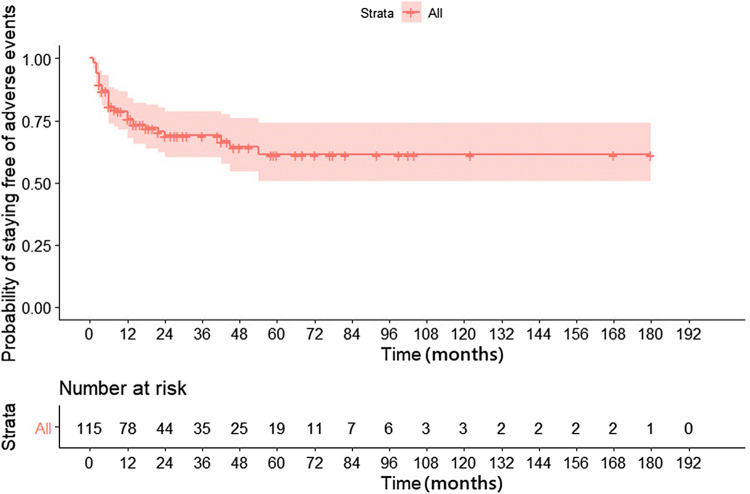
Kaplan Meier graph comparing treatment success between colonic strictures and small bowel strictures.

**Figure 3:**
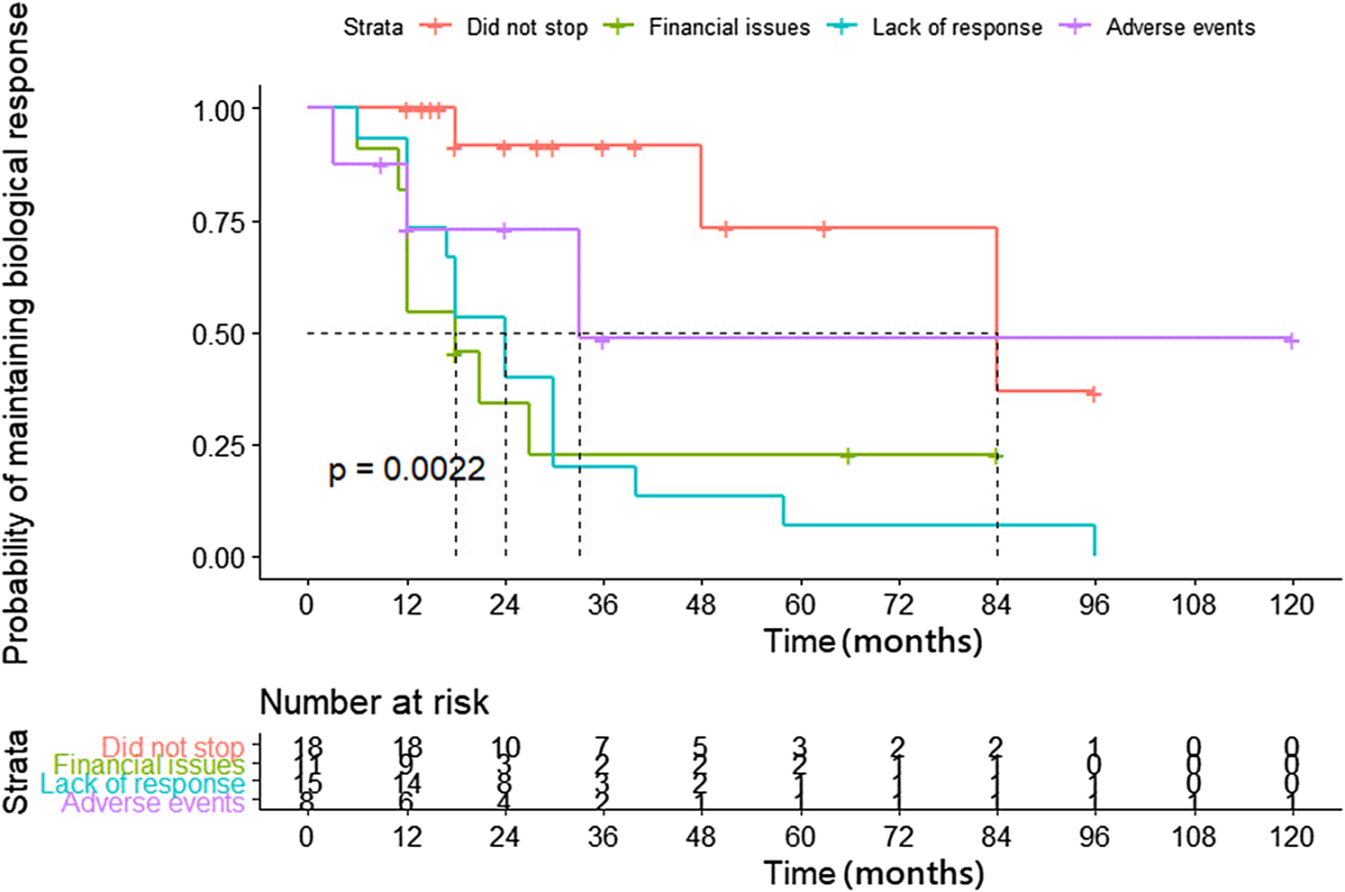
Kaplan Meier graph showing probability of maintaining response after anti-TNF discontinuation due to various reasons compared with patients continued to take anti-TNF.

Among fifty-nine patients, majority received infliximab (84.7%; n = 50) as initial therapy and rest (15.3%; n = 9) received adalimumab. The median duration of anti TNF therapy was 14 (range 2–96) months. 54.2% (n = 32) patients received concomitant therapy with immunomodulators. After induction therapy 88% (n = 52) patients had clinical improvement (Primary non response: 11.8%). Maintenance therapy was continued in 46 patients of whom 52.2 (n = 24) had a secondary loss of response at a median duration of 21 months. The overall cumulative probability of treatment success at the end of 1, 2 and 5 years was 69%, 51%, and 28% respectively (Table 2 & Fig. 1). Among patients who had response after induction therapy the cumulative treatment success rate at 1, 2 and 5 years was 78.5%, 59%, and 32.8% respectively. There was no difference between patients with colonic strictures versus small bowel strictures in the probability of maintaining response to biologics (Fig. 2). At the end of follow up (median of 42 months), 69.5% (n = 41) stopped biologics. Most common reason for stopping biologics was lack of response (37.3%; n = 22), followed by lack of finances (18.6%, n = 11), and severe adverse events (13.6%, n = 8). Those patients who stopped biologics due to lack of response had higher rate of surgery, compared to those who stopped due to other indications (9 vs 1; p = 0.01). However, the hospitalization rate and subsequent steroid requirement were similar between both groups. The overall probability of maintaining response was high in patients who continued to take biologics compared to those who stopped (Fig. 3). Among patients who stopped therapy, 11.8% (n = 7) switched to alternate anti-TNF therapy, 11.8% (n = 7) underwent surgical resection, 20.3% (n = 12) were maintained on immunomodulators (azathioprine/6-mercaptopurine/methotrexate), and 16.9% (n = 10) received partial exclusive enteral nutrition (EEN).

**Table 2:** Outcomes after initiation of biological therapy in stricturing Crohn’s disease.

Outcome	N (%)
**Initial biologic therapy**	
IFX	50 (84.7%)
ADA	9 (15.3%)
Cumulative success rate at 12 months	**69%**
Cumulative success rate at 24 months	**51%**
Cumulative success rate at 60 months	**28%**
Median duration of therapy (range) (months)	14 (2–96)
Primary non response	7 (11.8%)
Secondary loss of response	24 (52.2%)
Median duration of response after induction therapy	21 (12–38)
Biologics stopped	41 (69.5%)
Reasons for stopping biologics	
Non-affordability	11 (18.6%)
Lack of response	22 (37.3%)
Adverse events	8 (13.6%)
**Therapy offered in patients who stopped biologics**	
Switch to another biologic	7 (11.9%)
Maintained on immunomodulators	12 (20.3%)
Surgery	7 (11.8%)
EEN	10 (16.9%)

Among the factors predicting treatment success with anti-TNF therapy, anaemia at presentation was the only clinical factor which predicted poor response on univariate analysis, rest of the factors including age at onset less than 30 years, male sex, intestinal obstruction, perianal fistula, EIM, colonic stricture, steroid dependency and early initiation of biologics, were not significant on cox regression analysis (Table 3). Colonic strictures, anaemia at presentation and early initiation of biologics were included in multivariate analysis, but they were not significant. Factors predicting primary non-response and secondary loss of response were also analysed. Only female sex predicted primary non-response on univariate analysis and none of the factors predicted secondary loss of response (supplementary table 1 & 2).

**Table 3:** Factors predicting anti-TNF failure in stricturing Crohn’s disease.

Factor	Univariate		Multivariate	
	HR (Confidence intervals)	*P* value	HR (Confidence intervals)	*P* value
Age at onset less than 30 years	0.9 (0.4–1.9)	0.8	–	–
Male sex	0.9 (0.3–2.1)	0.9	–	–
Intestinal obstruction	0.8 (0.2–2.7)	0.7	–	–
Perianal fistula	0.8 (0.3–2.1)	0.8	–	–
Extra intestinal manifestations	1.3 (0.5–3.3)	0.6	–	–
Colonic strictures	0.5 (0.2–1.2)	0.1	0.5 (0.2–1.3)	0.1
Anemia at presentation	2.1 (1.0–4.6)	0.05	1.8 (0.9–4.0)	0.09
Steroid dependent disease	0.8 (0.3–1.6)	0.5	–	–
Early initiation of biologics	0.4 (0.2–1.0)	0.07	0.4 (0.2–1.0)	0.08
Concomitant IM	1.2 (0.6–2.7)	0.5	–	–

### Adverse events

A total of 16.9% (n = 10) adverse events occurred. 5.1% of patients had reactivation of tuberculosis, 1.7% had infusion reactions requiring temporary cessation of infusion. Two (3.4%) developed viral infections including one patient with herpes zoster and one patient with varicella infection. One patient developed skin rash which required therapy discontinuation. (Table 4).

**Table 4:** Adverse events secondary to biologics.

Adverse event	N (%)
Tuberculosis reactivation	3 (5.1%)
Pneumonia	1 (1.7%)
Skin rash requiring withdrawal	1 (1.7%)
Urticaria	1 (1.7%)
Infusion reaction	1 (1.7%)
Bacterial sepsis	1 (1.7%)
Zoster	1 (1.7%)
Varicella	1 (1.7%)

## Discussion

The present study demonstrates the efficacy of anti-TNF therapy in patients with stricturing CD, a patient population which is challenging to treat medically. 69% of patients in this cohort had treatment success at 12 months without the requirement for any treatment change or additional therapy. This success rate, however, fell to 28% at 5 years, primarily due to loss of response, and to a lesser extent due to treatment withdrawal because of adverse events or prohibitive cost. Majority were difficult to treat, and were either steroid dependent or steroid refractory, had prolonged disease duration (median duration of disease 10 years), and ~ 2/3^rd^ had multiple strictures. Among the available immunosuppressive therapies, steroids can reduce inflammation and mucosal oedema and are considered in case of active obstructive symptoms, but their role in mucosal healing and resolution of stricture has not been demonstrated, and they are not advised for maintenance. Among biologics, anti-TNF agents are considered as treatment of choice in the presence of inflammation.

The results of the present study are similar to the largest, multicentre, prospective study which evaluated efficacy of adalimumab in 97 patients with small bowel stricturing CD (CREOLE). In this study, 64% of patients with symptomatic strictures had treatment success at 24 weeks whereas, at a median follow up of 3.8 years, the treatment was successful only in 29%. Overall, 50% of patients did not require bowel resection 4 years after study inclusion^5^. In the present study majority of patients (84.7%) received infliximab and the outcome measures were similar to CREOLE study. The treatment success at 12 months was almost similar in both studies (61% vs 69%), and the cumulative probability at 5 years was also similar to the long-term results of the CREOLE study (25% vs 29%). In our cohort, only 11.8% patients underwent surgery, as compared to the CREOLE study where > 50% patients required resection. More than 40% patients in CREOLE study had previous intestinal resection (which is the strongest risk factor for surgery), which could explain the higher rates of surgery in the CREOLE study. Moreover, most of the patients in our study had multiple strictures, which precluded surgery because of the high risk of developing short bowel syndrome with resection. Another prospective study by Pallotta et al., demonstrated 53% resolution of small bowel strictures with infliximab using small bowel ultrasound^7^. But these findings were not replicated in another prospective study by Condino et al., in which there was no significant change in sonographic parameters after infliximab or adalimumab therapy^8^. Resolution of strictures was not evaluated in the present study, because of lack of expertise in small bowel USG, and logistics with repeating cross-sectional imaging to evaluate stricture resolution.

Endoscopic dilatation was done in only 5% in our study. This was because of the fact that majority in our patients had multiple inflammatory strictures located in the small bowel which were not suitable for dilatation. Recent patient level meta-analysis showed that even though there is 80% clinical improvement after EBD, the effect was short term and two thirds required repeat dilatation and one third required surgery^17^ At follow-up, even though overall retention rate of biologics was low in our study (69.5% stopped biologics), approximately 50% of patients stopped because of lack of finances or adverse events. Interestingly, the long-term probability of response was higher among the patients who stopped because of lack of finance or adverse events than those who stopped because of loss of response.

Among the predictors of response to anti-TNF therapy, pre-stenotic dilatation and long segment strictures have been consistent across the studies which predict high risk of surgery^18,19^ We did not evaluate the radiological predictors in this study, and among the clinical predictors anaemia at presentation was the only factor associated with poor response to anti-TNF therapy. In a retrospective study from Japan, among 53 patients with small bowel strictures, half required surgery at 5 years, and pre-stenotic dilatation and long segment stricture were associated with higher while immunomodulator or biologic usage was associated with lower risk of surgery^20^. In a real-life cohort study from Italy which included 51 patients, 39% required surgery during follow up, and presence of colonic and ileocolonic location was associated with increased risk of surgery^21^ unlike our study presence where stricture location was not associated with treatment failure.

The literature on the role of anti-TNF therapy in reducing the rates of surgery in CD remains uncertain. Observational studies suggest that early initiation of biologics prevent disease progression from inflammatory phenotype to stricture development. A retrospective study from the Pediatric Inflammatory Bowel Disease Collaborative Research Group registry involving 1442 paediatric IBD patients showed early initiation (< 3 months of diagnosis) of biologics was associated with avoidance of surgery but it was evident only after 5 years^22^. Among adult patients, Swiss IBD cohort showed among large group of patients that early treatment (< 2 years) with IM or biologics was associated with decreased intestinal surgery, perianal surgery, and stricturing complications^23^.

In our study infective complications were seen in 11.8% (n = 7) patients, among them TB reactivation was seen in three, and viral infections were seen in two patients. Overall, 8 patients had to stop biologics due to adverse events. All patients who developed TB reactivation had been screened for latent TB before initiation of anti TNF therapy. Incidence of TB reactivation following anti-TNF therapy is high in TB endemic areas as shown in recent meta-analysis^24^.

The major limitation of our study are its retrospective design and small sample size. Further, the response assessment was based only on clinical parameters rather than objective demonstration of improvement with endoscopy or radiology. Response assessment in stricturing CD is inconsistent across studies with most studies looked at risk of surgery but it will be influenced by indication for surgery, decision making threshold of treating physician and patient preference. Lack of therapeutic drug monitoring is also another major limitation in our study. However, there are no well-defined cut offs of trough levels to be targeted in patients of stricturing CD. This phenotype may need higher levels as in the case of fistulizing CD especially perianal fistulae which needs to be further explored.

## Conclusion

Stricturing Crohn’s disease is a challenging problem to manage and anti TNF therapy is associated with good short-term treatment success and a modest long-term response. Anaemia at presentation predicts poor response to anti-TNF therapy.

## Supplementary Information

Supplementary Information.
