# Supplementary material for: Real world analysis on the efficacy and safety of anti-tumor necrosis factor therapy in patients with stricturing Crohn’s disease
Source: Sci Rep. 2021 Jun 3;11:11704. doi: 10.1038/s41598-021-90660-2 (PMC8175366; doi:10.1038/s41598-021-90660-2)
Supplement: Supplementary file 1 — Supplementary Information. [file 41598_2021_90660_MOESM1_ESM.docx]

Supplementary table 1: Factors predicting primary non response

| Factor | Univariate | | Multivariate | |
| --- | --- | --- | --- | --- |
|  | HR (Confidence intervals) | P-value | HR (Confidence intervals) | P-value |
| Age at onset less than 30 years | 0.5 (0.1-2.3) | 0.3 | - | - |
| Male sex | 0.1 (0.03-0.8) | 0.03 | - | - |
| Intestinal obstruction | 1.4 (0.1-11) | 0.7 | - | - |
| Perianal fistula | 0.03 (0.0-226) | 0.4 | - | - |
| Extra intestinal manifestations | 0.7(0.0-5.9) | 0.7 | - | - |
| Colonic strictures | 0.03 (0.08-75.2) | 0.3 |  | - |
| Anemia at presentation | 1.7 (0.3-9.0) | 0.5 |  | - |
| Steroid dependent disease | 2.0 (0.4-10.7) | 0.3 | - | - |
| Early initiation of biologics | 0.3 (0.04-3.0) | 0.3 |  | - |
| Concomitant IM | 0.6 (0.1-2.7) | 0.5 | - | - |

Supplementary table 2: Factors predicting secondary loss of response

| Factor | Univariate | | Multivariate | |
| --- | --- | --- | --- | --- |
|  | HR (Confidence intervals) | P-value | HR (Confidence intervals) | P-value |
| Age at onset less than 30 years | 0.8 (0.3-1.9) | 0.7 | - | - |
| Male sex | 0.8 (0.3-2.0) | 0.6 | - | - |
| Intestinal obstruction | 0.6 (0.1-2.6) | 0.5 | - | - |
| Perianal fistula | 1.0 (0.4-2.7) | 0.8 | - | - |
| Extra intestinal manifestations | 1.7 (0.6-4.6) | 0.2 | - | - |
| Colonic strictures | 0.6 (0.2-1.6) | 0.3 |  |  |
| Anemia at presentation | 1.8 (0.8-4.2) | 0.1 |  |  |
| Steroid dependent disease | 0.8 (0.3-1.9) | 0.7 | - | - |
| Early initiation of biologics | 0.6 (0.2-1.4) | 0.2 |  |  |
| Concomitant IM | 1.8 (0.7-4.2) | 0.1 | - | - |
